# Endotracheal metastasis of hepatocellular carcinoma: a case report

**DOI:** 10.1186/s40248-019-0182-7

**Published:** 2019-06-06

**Authors:** Giacomo Ghinassi, Pasquale Imitazione, Alfonso Pecoraro, Luciano B. G. Montella, Paola Martucci, Raffaella Giacobbe, Campione Severo, Domenico Aronne

**Affiliations:** 1 Dipartimento di Medicina Clinica e Chirurgia, Sezione di Malattie dell’Apparato Respiratorio, Università Federico II, Monaldi hospital, via L. Bianchi, 80131 Naples, Italy; 2 Dipartimento Onco-pneumo-ematologico, Servizio di Endoscopia Bronchiale ed Urgenze broncologiche, Ospedale Antonio Cardarelli, Naples, Italy; 3 Dipartimento di Anatomia Patologica, Ospedale Antonio Cardarelli, Naples, Italy

## Abstract

We describe the case of a 75 years old patient with a history of hepatocellular carcinoma, with acute respiratory failure due to tracheal obstruction by metastasis, successfully treated with airway disobstruction with rigid bronchoscope.

## Case report

**Fig. 1:**
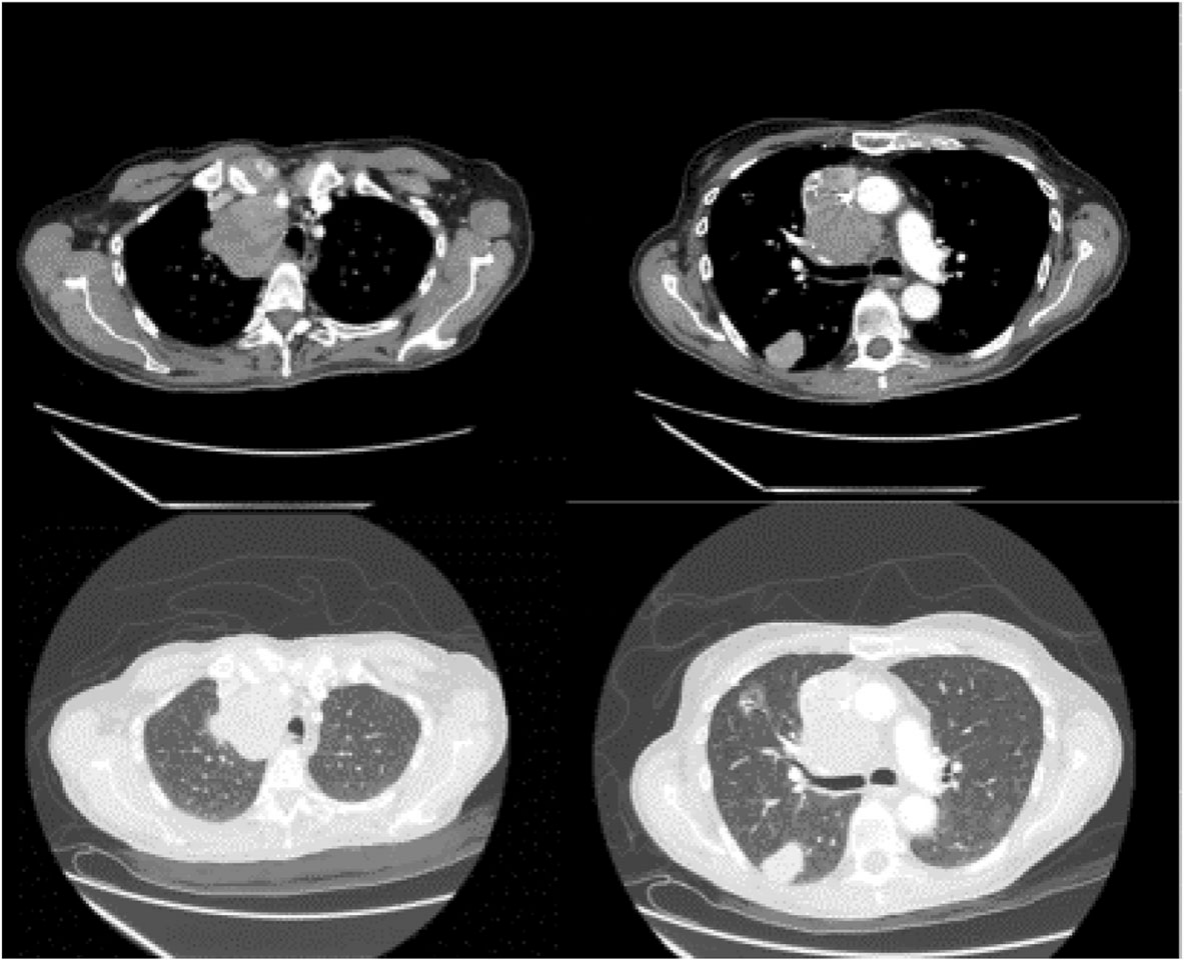
Chest enhanced computed tomography (CT) showed a solid lesion in the apical segment of right lower lobe with multiple confluent mediastinal adenopathies and right paratracheal lymphadenopathy

A 75 years old man, smoker, with a past history of a hepatic transplantation 13 years earlier for a hepatocellular carcinoma, was admitted to hospital with hemoptysis and dyspnea. He performed a chest CT scan, showing a solid lesion in the apical segment of right lower lobe with multiple confluent mediastinal adenopathies and right paratracheal lymphadenopathy (Fig. 1).

We practiced a videobronchoscopy that showed two small sessile lesions approximately 4.5 cm far from the carina on the right lateral wall of the trachea, which were removed with biopsy forceps. EBUS-TBNA was performed on the right paratracheal lymph node. The pathological findings were suggestive for hepatocarcinoma metastases and the patient was underwent chemotherapy.

**Fig. 2:**
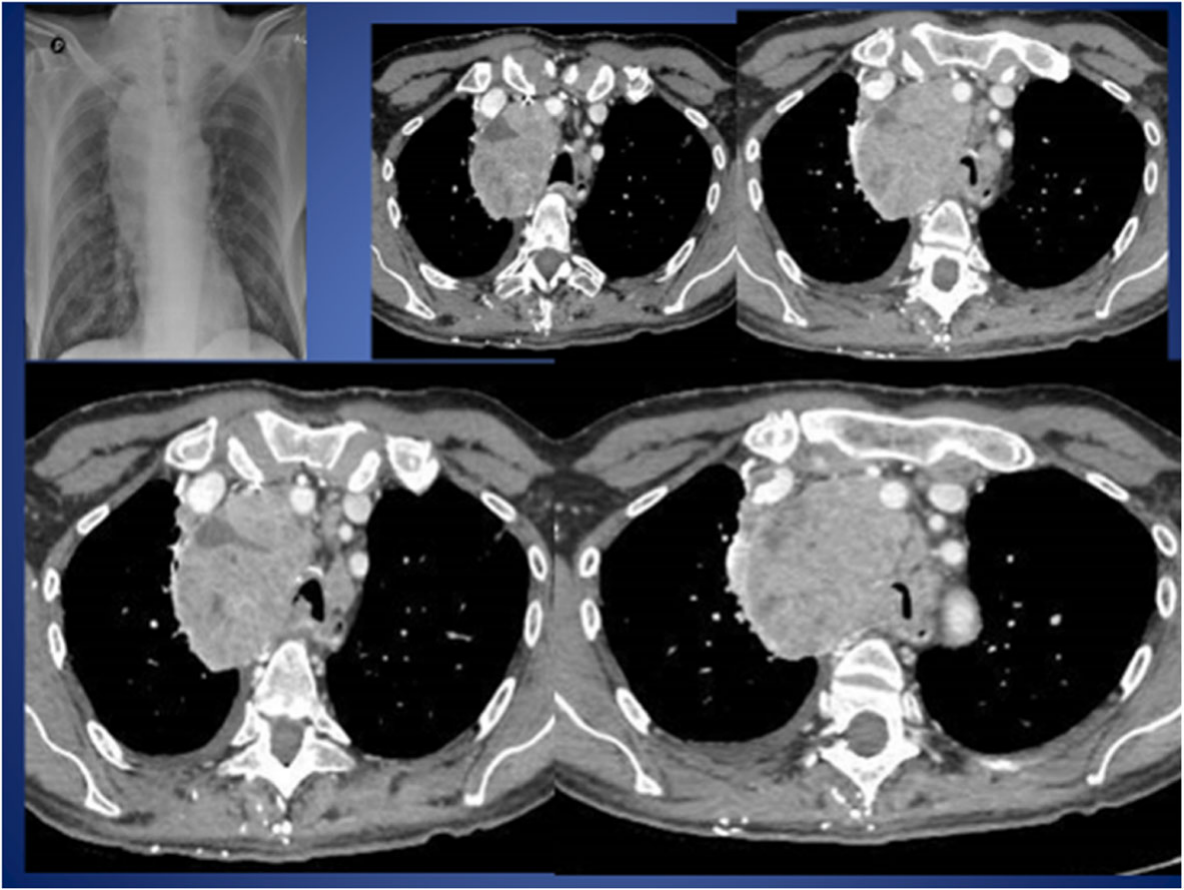
Chest radiography and Computed Tomography (CT) showed a solid neoplastic lesion in the apical segment of the right lower lobe and significant stenosis of the tracheal lumen

After six months, the patient returned to the emergency room for wheezing and acute respiratory failure. Chest x-ray and CT scan showed deterioration of the radiological picture with stenosis of the tracheal lumen (Fig. 2).

**Fig. 3:**
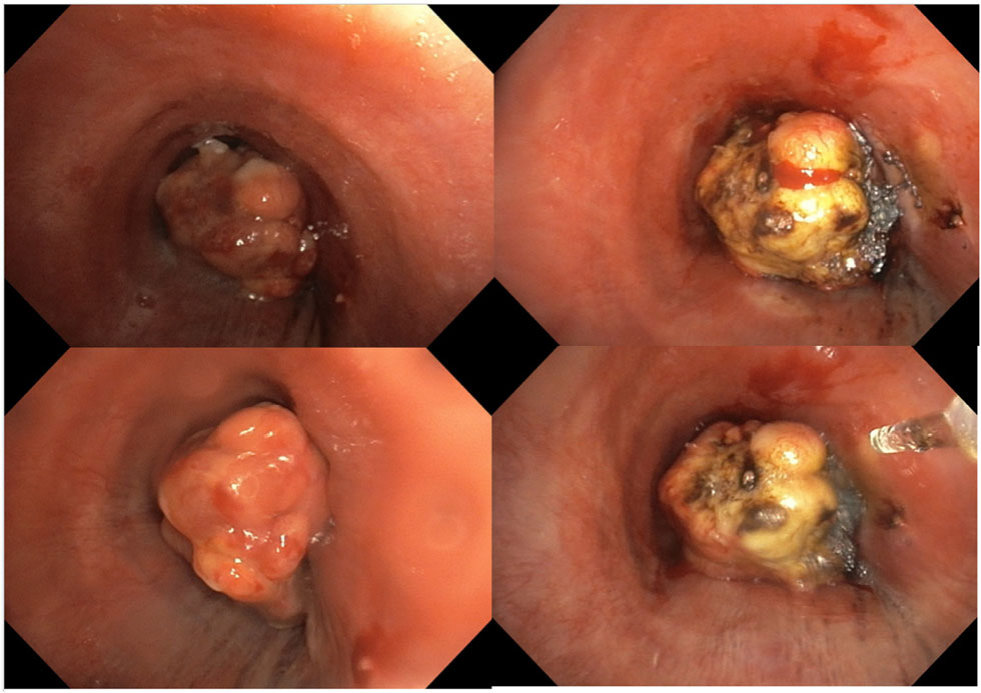
Bronchoscopy revealed that the tumor completely obstructed the tracheal lumen

**Fig. 4:**
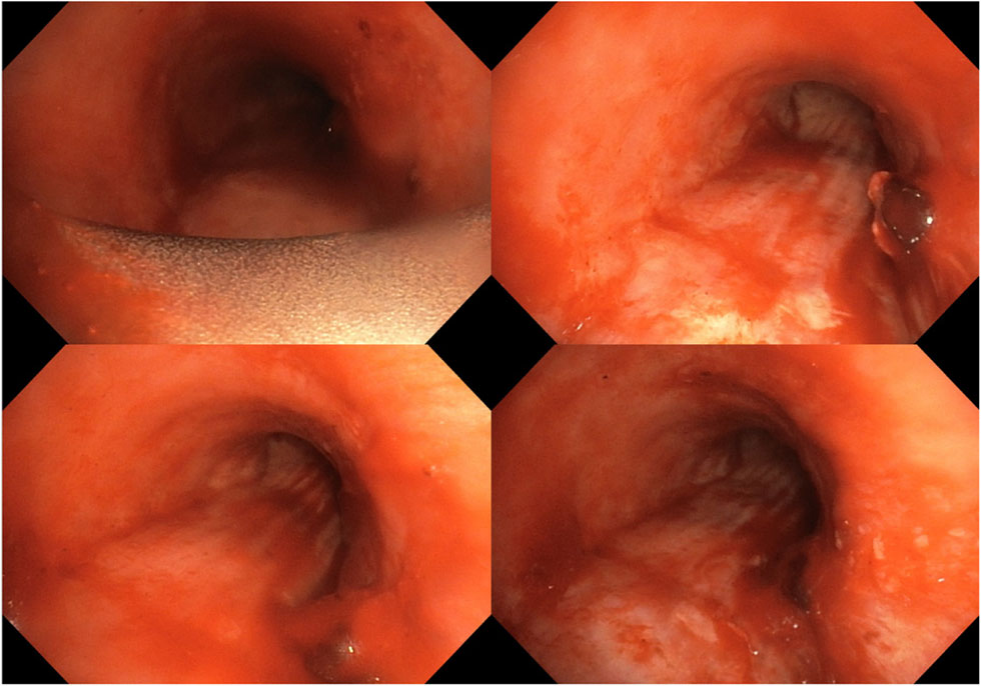
After bronchial disobstruction, bronchoscopy revealed the recanalization of tracheal lumen

**Fig. 5:**
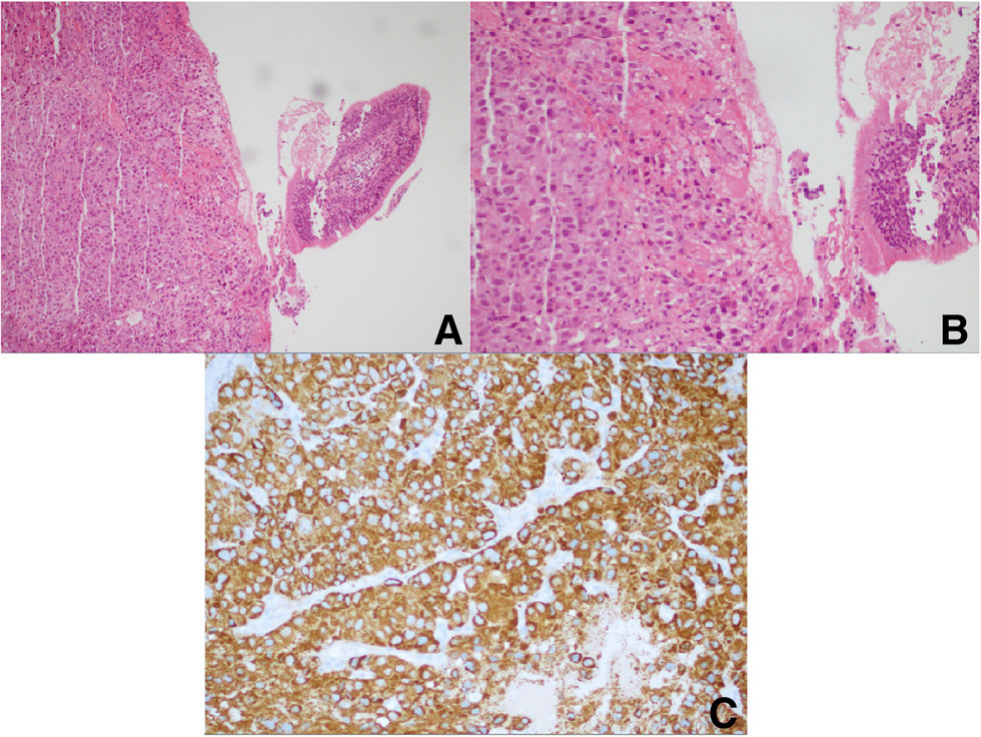
**a**) 100 enlargements, hematoxylin and eosin. Solid-growth non-small cell epithelial neoplasia. On the right fragment of ciliated cylindrical epithelium of the respiratory tract; **b**) 200 enlargements, same field; **c**) 200 enlargements, immunohistochemical anti-hepatocyte antigen: diffuse cytoplasmic granular positivity according to the hepatic origin of the neoplasia (hepatocellular carcinoma)

The patient made videobronchoscopy that showed a vegetative neoformation which obstructed the tracheal lumen about 6.5 cm far from the true vocal cords (Fig. 3). The patient was intubated with a rigid bronchoscope Storz n°14 and we used laser photocoagulation to devascularize the lesion that was subsequently removed with a debulking maneuver, recanalizing the trachea (Fig. 4). The anatomopathological findings confirmed the previous diagnosis of hepatocarcinoma metastases (Fig. 5).

## Discussion and Conclusion

The interest of this case is essentially due to two reasons: the rarity of the metastatic localization of hepatocellular carcinoma, which, as reported in the literature, has an incidence of 0.04% [1] and the importance of rigid bronchoscopy in the resolution of respiratory failure secondary to tracheal obstruction.

The lungs represent the most common site of liver metastases, reported in the 37–70% of cases at autopsy but less often clinically detected. These appear as nodules, often multiple and pleural effusion is common. Many nodules have the tendence to appear in the right-lower lobe, and the greatest degree of effusion occurs in the lower lobes, suggesting a probable transdiaphramatic spread. Occasionally, these metastases spread and give a miliary pattern [2]. Another possible mechanism is lymphatic spread, as probably happened to our patient. In fact mediastinal lymph nodes were involved since the tracheal lesion appeared.

Tracheal localization appears particularly important for the risk of incurring acute respiratory failure, as happened to our patient. Rigid bronchoscopic therapy is required for the treatment of patients with central airway obstruction. Various bronchoscopic techniques are available for tracheobronchial tumors, including neodymium-yttrium-aluminum-garnet (Nd-YAG) laser therapy, electrocautery, brachytherapy, photodynamic therapy, cryotherapy, and APC [3].

In conclusion, interventional bronchoscopy in most cases of acute airway obstruction from cancer is palliation, not cure. Reestablishment of patient airways may avoid hospitalization in a critical care unit, prolonged intubation and mechanical ventilation, and enhances patient’s ability to accept and undergo systemic chemotherapy, immunotherapy or radiation therapy [4]. It also determines an immediate symptomatic relief and an improvement in the quality of life [5, 6].

## Abbreviations

APC: Argon Plasma Coagulation
CT: Computed Tomography
EBUS-TBNA: Endobronchial Ultrasound Transbronchial Needle Aspiration
